# Reduction of Airway Hyperresponsiveness by KWLL in *Dermatophagoides-pteronyssinus*-Challenged Mice

**DOI:** 10.1155/2013/262391

**Published:** 2013-03-04

**Authors:** Chih-Che Lin, Shulhn-Der Wang, Li-Jen Lin, Hong-Jye Hong, Chin-Jen Wu, Chung-Jen Chiang, Yun-Peng Chao, Shung-Te Kao

**Affiliations:** ^1^Graduate Institute of Chinese Medicine, China Medical University, Taichung 40402, Taiwan; ^2^School of Post-Baccalaureate Chinese Medicine, College of Chinese Medicine, China Medical University, Taichung 40402, Taiwan; ^3^School of Chinese Medicine, College of Chinese Medicine, China Medical University, Taichung 40402, Taiwan; ^4^Department of QC/R&D, Kaiser Pharmaceutical Co., Ltd., Tainan 71041, Taiwan; ^5^Department of Medical Laboratory Science and Biotechnology, China Medical University, Taichung 40402, Taiwan; ^6^Department of Chemical Engineering, Feng Chia University, Taichung 40724, Taiwan; ^7^Department of Chinese Medicine, China Medical University Hospital, Taichung 40402, Taiwan

## Abstract

Urine therapy has been commonly practiced in ancient civilizations including those of India, China, and Greece. The traditional Chinese medicine KWLL, the precipitation of human urine, has been used in China to alleviate the symptoms of asthma for thousands of years. However, the mechanism of action by which KWLL exerts its immunotherapy is unclear. This study attempted to elucidate the pharmacology of KWLL in mice that had been challenged recurrently by *Dermatophagoides pteronyssinus* (Der p). BALB/c mice were orally administered KWLL (1 g/kg) before an intratracheal (i.t.) challenge of Der p. Allergic airway inflammation and remodeling were provoked by repetitive Der p (50 **μ**g/mice) challenges six times at 1 wk intervals. Airway hypersensitivity, histological lung characteristics, and the expression profiles of cytokines and various genes were assessed. KWLL reduced Der p-induced airway hyperresponsiveness and inhibited eosinophil infiltration by downregulating the protein expression of IL-5 in bronchoalveolar lavage fluid (BALF). It also inhibited neutrophil recruitment by downregulating IL-17A in BALF. KWLL effectively diminished inflammatory cells, goblet cell hyperplasia, and mRNA expression of IL-6 and IL-17A in the lung. The reduction by KWLL of airway inflammatory and hyperresponsiveness in allergic asthmatic mice was mediated via immunomodulation of IL-5, IL-6, and IL-17A.

## 1. Introduction

The incidence of asthma has increased dramatically, along with its morbidity and mortality. It is now a common chronic disorder in the world, especially in developed countries, and 255,000 people died of asthma in 2005 [1, 2]. The prevalence of asthma has increased approximately 3-fold in children over the past few decades, and 300 million people are now affected worldwide [3]. However, the diagnosis and management of asthma are more difficult than other chronic diseases because asthma patients have complex heterogeneous syndromes and pharmacological responses to medicine [4]. Most patients with asthma use a combination of steroids and *β*2-adrenergic agonists as the standard therapy [5, 6]. Unfortunately, patients who inhaled these drugs over the long term can develop serious side effects, and some patients do not satisfactorily respond to these drugs [7]. 

Therefore, an increasing number of patients have sought complementary and alternative medicine (CAM), such as traditional Chinese medicine (TCM), herbal products, acupuncture, breathing techniques, and homeopathy [8]. Recently, several publications have shown that the TCM therapies used for asthma are safe and effective [9]. According to the *Shang Han Lun *which was written by Zhang Zhong Jing (150–219), KWLL, a TCM made from the sediment of human urine, has been applied for the treatment of syndromes related to lung diseases [10]. Moreover, there are also many records of the practice of urine therapy from different cultures from the Middle Ages to recent times, and those countries have referred to the urine as “gold of the blood” and an “elixir of long life” [11]. However, the regular mechanisms of KWLL in asthma remain unclear.

Allergic asthma results from serial and repeated inflammatory processes that involve numerous types of white cells, mast cells, and macrophages, which infiltrate into lung tissue, and these cells secrete many different cytokines, chemokines, and growth factors [12]. Overall, these cells' and mediators' interactions and functions in asthma patients result in the extraordinary lung structures and dyspnea that compose the basic characteristics of asthma [13].In particular, the IL-17A, a secretion cytokine of T_H_17 cells, increases contractility of airway smooth muscle which contributes to allergen-induced airway hyperresponsiveness in asthma [14].

We previously developed a mouse model of allergic asthma that exhibits symptoms similar to those in patients with allergic asthma, including high levels of IgE in the serum, airway hyperresponsiveness, and airway inflammation and remodeling [15]. We used this mouse model to investigate the possible immunoregulatory effects of KWLL on asthma.

## 2. Materials and Methods

### 2.1. Mice and Reagents

The experiments on mice were approved by the Institutional Animal Care and Use Committee of the China Medical University (no. 100-138-N), and all of the mouse treatments were in accordance with the guidelines of the National Science Council of the Republic of China. Five-week-old male mice that were specifically pathogen-free of BALB/c were purchased from the National Laboratory Animal Center, Republic of China. Crude Der p was prepared as previously described [15].

### 2.2. KWLL Preparation

**Figure 1:**
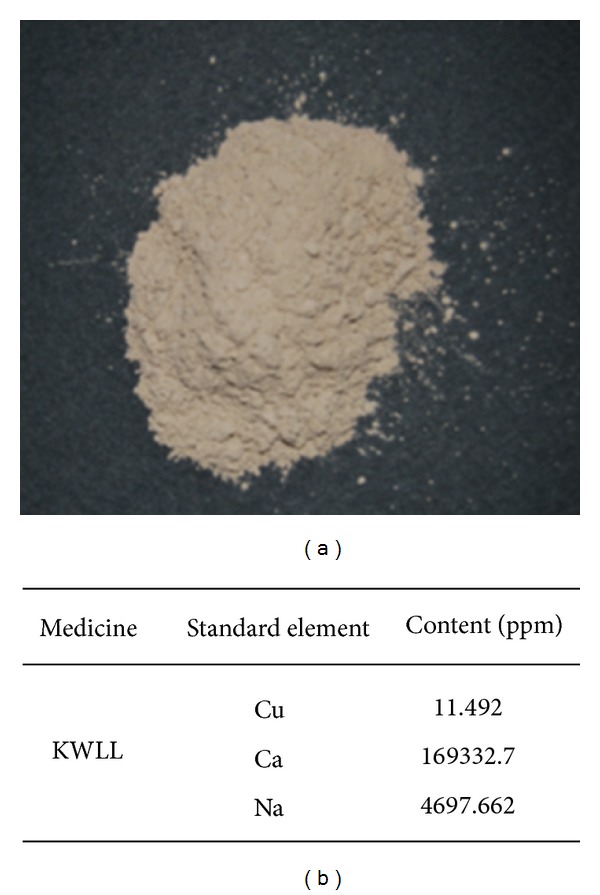
(a) Picture of KWLL. (b) The standard elements in KWLL.

KWLL powder (Figure 1(a)) was collected from the precipitation of urine of elementary school children who were aged between 7 to 12 years old of boys and identified by Kaiser Pharmaceutical Co., Ltd. (GMP Pharmaceutical Company, Tainan, Taiwan). 1 g KWLL was produced from about 10–18 L urine. KWLL was dissolved in distilled water for administration in mice and stored at −20°C before use.

### 2.3. Microwave-Assisted Acid Digestion

A microwave-accelerated reaction system (MARSXpress, CEM Corporation, Matthews, NC, USA) was used to digest the acidified samples of KWLL. The full power of the system was approximately 1200 W of microwave energy at a frequency of 2.45 GHz. The system was equipped with a 40-vessel turntable. The instrument was precalibrated according to the manufacturer's specifications. The parameters of the microwave system were 600 W of power (100%), heated to 175°C over 5.5 min, maintained for 4.5 min, and then cooled for 1 h.

### 2.4. Inductively Couple Plasma-Optical Emission Spectrometry

A sequential Jobin Yvon ULTIMA 2000 spectrometer operating at a forward power of 1 kW and equipped with a Meinhard-type nebulizer and Scott spray chamber was used. The flow rates were as follows: plasma gas, 12 L/min; sheath gas, 0.2 L/min; nebulizer gas, approximately 0.6 L/min (optimized each day); and sample introduction, 1 mL/min. The wavelength for Cu was 324.754 nm; for Ca, the wavelength was 396.847 nm; and for Na, the wavelength was 568.821 nm. KWLL contains these three standard elements (see Figure 1(b)).

### 2.5. Allergen Challenge and Assessment of Airway Inflammation

Allergic airway inflammation and remodeling were provoked by repetitive to Der p (1 mg/mL, 50 *μ*L/mice) in PBS challenges once a week for 5 weeks (total 6 doses). In the Der p group, BALB/c mice (*n* = 6) were orally administered water and challenged with intratracheal Der p. In the KWLL group, mice were orally administered KWLL (1 g/kg) 30 min before inoculation with Der p. In parallel experiments, PBS group were orally administered water and intratracheally administered PBS. Mice were sacrificed by i.p. injection of xylazine (200 *μ*g/mice) and ketamine (2 mg/mice) three days after the last challenge as reported previously [15, 16]. BALF and serum were stored at −80°C until further assays. After total leukocyte counting, differential counts of white cells were measured with a Liu stain (Biotech, Taiwan) in a blind manner. 

### 2.6. Measurement of Airway Hyperresponsiveness

Airway responsiveness was measured according to the manufacture's protocol (Buxco Electronics, Inc., Troy, NY, USA) in response to methacholine induction as previously described [15].

### 2.7. Histology of Lung Specimens

Paraffin-embedded tissues were sectioned at 5-*μ*m and stained with PAS and H&E stains, respectively. The identification of tissue sites was determined at 400x magnification. The degree of the airway inflammation was exhibited as inflammatory cell infiltration. A five-point scoring system (grade 0 to 4) was used as follows: grade 0 (no inflammatory cells); grade 1, <25%; grade 2, 25%–50%; grade 3, 50%–75%; grade 4, ≥75%; and grade 5, ≥75% [17].

### 2.8. Collagen Analysis

Collagen extracted from 100 mg of the lung tissue of mice was homogenized in liquid nitrogen. The supernatant of extraction was assayed with a collagen assay kit (Biocolor, Belfast, UK).

### 2.9. Flow Cytometric Analysis

PerCP-conjugated antimouse CD3, PE and/or FITC-conjugated antimouse CD4, FITC-conjugated antimouse CD8, and FITC-conjugated antimouse CD25 (BD Pharmingen) were used for FACScan staining. After the BALF cells (1 × 10^5^) were stained, the stained cells were analyzed with FACScan (Becton-Dickinson Immunocytometry System, San Jose, CA, USA).

### 2.10. Measurement of Der p-Specific IgG1, IgG2a/2b and Total IgE

Serum samples of total IgE (1 : 2 dilution) and Der p-specific IgG1 and IgG2a/2b (1 : 4 dilute) were measured by ELISA (BD Pharmingen, San Diego, CA, USA). Der p-specific IgG1 and IgG2a/2b were detected using biotin antimouse IgG1 and IgG2a/2b Abs (BD Pharmingen, San Diego, CA, USA) and the results are expressed as OD_450_. 

### 2.11. ELISA of Cytokine Levels

The amounts of cytokines IL-5, IL-6, IL-12, and IL-17A were determined using a commercially available ELISA Ready-SET-Go! Kit (eBioscience, San Diego, CA, USA). The IL-13 and IFN-*γ* levels were assayed with an ELISA DuoSet! Kit (R&D System, Abingdon, UK). The reaction was used to detect cytokine concentrations according to the manufacturer's protocol. 

### 2.12. Quantitative Real-Time Polymerase Chain Reaction (qPCR)

Total RNA was extracted by a Trizol reagent following the Invitrogen Life Technologies protocol. Total RNA samples were reverse-transcribed to cDNA using a High Capacity cDNA Reverse Transcription Kit (Applied Biosystems, Foster City, CA, USA). qPCR was conducted with 1 *μ*L of cDNA, a FastStart Universal SYBR Green Master kit (Roche), and a specific gene primer set as reported previously [15].

### 2.13. Statistical Analysis

The results are shown as the means ± standard errors. The Student's *t*-test was used to estimate the differences in the values between the groups. A value of *P* < 0.05 was considered significant. 

## 3. Results

### 3.1. KWLL Suppressed Der p-Induced Airway Hyperresponsiveness in the Asthmatic Mouse Model

**Figure 2:**
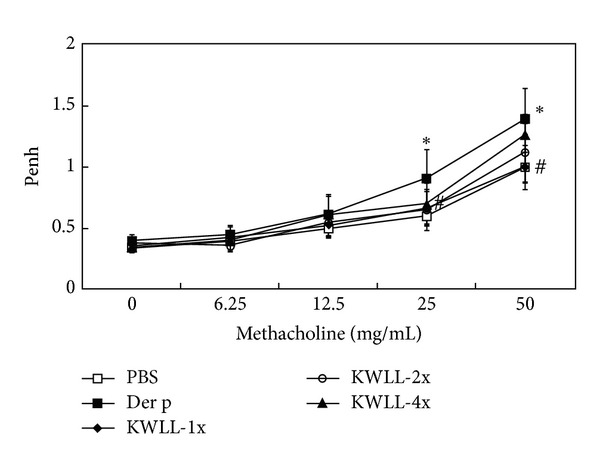
The effects of KWLL on Der p-induced airway hyperresponsiveness were determined on day 3 after the last challenge. Values represent the means ± SDs of 6 mice. *P < 0.05 (versus PBS group);  ^#^ *P* < 0.05 (between nontreated and KWLL-treated groups).

First, we used a repetitive Der p challenge mouse model to investigate the impact of oral KWLL on allergen-induced airway hyperresponsiveness. To determine which dose was the most suitable concentration and reduce animal sacrifice for subsequent experimentation, enhanced pause (Penh) values were used as the selection marker of airway responsiveness. Moreover, the mice were administered serially increasing doses of KWLL (1 g/kg: 1X, 2 g/kg: 2X, and 4 g/kg: 4X) 30 min before challenge with Der p. We found that the mice in the Der p group had higher Penh values than the PBS group, a difference that was particularly statistically significant at the maximum dose of methacholine. However, there was also a significantly greater decrease in Penh values in the 1X (1 g/kg) group than the Der p group at the maximal dose of methacholine (50 mg/mL) (Figure 2). 

### 3.2. KWLL Attenuated the Der p-Induced Airway Inflammation

**Figure 3:**
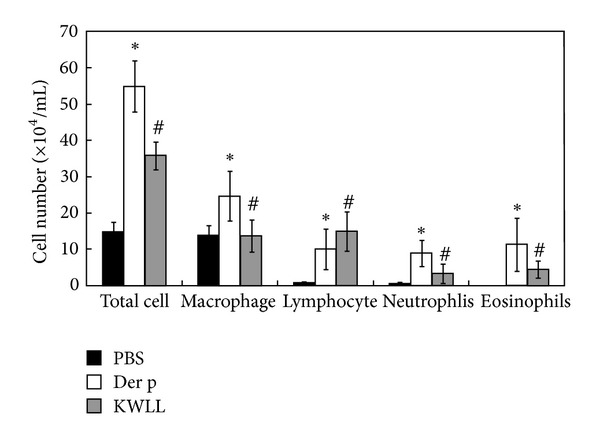
The effects of KWLL on Der p-induced airway inflammatory infiltrates in BALF. Mice received intratracheal instillation of Der p and were treated with KWLL or placebo (water). The total number of cells and the differential counts in BALF were assayed in the mice as described in Section 2. Values represent the means ± SDs of 6 mice. *P < 0.05 (versus PBS group); ^#^ *P* < 0.05 (between nontreated and KWLL-treated groups).

For subsequent study, the BALB/c mice were orally administered 1 g/kg KWLL at 1 wk intervals with each Der p inoculation, and the mice were sacrificed at 72 hr after the last challenge. In the PBS mice, eosinophils were not detected in BALF. However, in the Der p group, the total cells, macrophages, neutrophils, lymphocytes, and eosinophils in BALF were markedly higher than in the PBS mice (Figure 3). In the KWLL group, the total cells, macrophages, neutrophils, and eosinophils were significantly lower in BALF than the Der p group.

### 3.3. KWLL Improved Der p-Induced Lung Pathology

**Figure 4:**
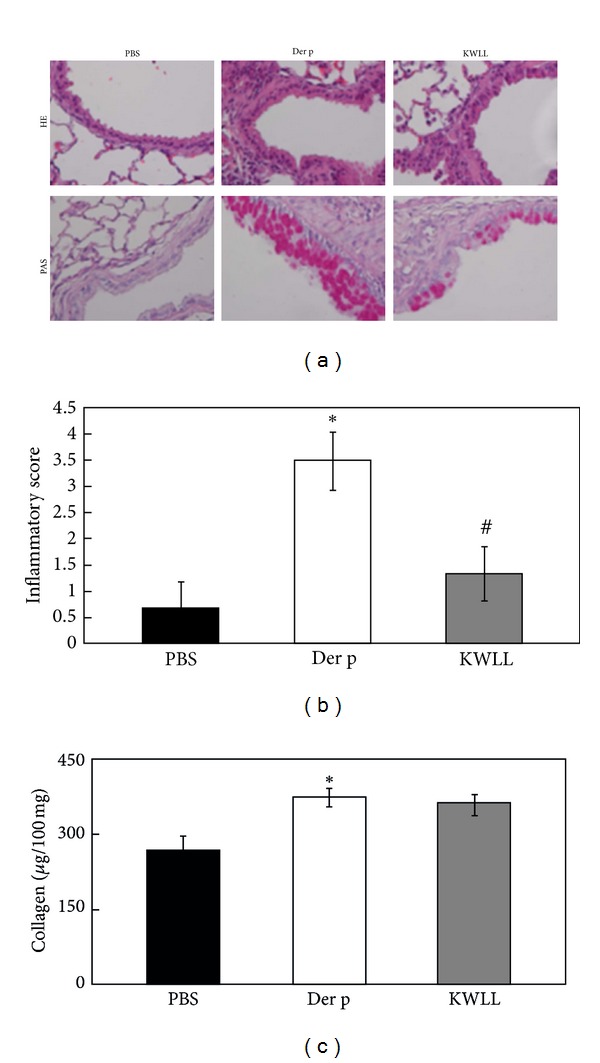
The Der p-induced lung morphology changes caused by KWLL treatment in mice. (a) Inflammation cells (top panel; H&E stain). (b) Goblet cell hyperplasia (bottom panel; PAS stain). Degrees of inflammation cells were estimated as described in Section 2. (c) Collagen was deposited in lung tissue. Values represent the means ± SDs of 6 mice. *P < 0.05 (versus PBS group); ^#^ *P* < 0.05 (between nontreated and KWLL-treated groups).

Airway inflammation, hyperplastic goblet cells, and collagen deposition are typical features of asthma patients. In the Der p group, the histological sections of lung tissue exhibited increased inflammatory cells and hyperplastic goblet cells (Figure 4(a)), compared with the PBS group. In contrast, the KWLL group showed significantly reduced inflammatory cells and hyperplastic goblet cells compared with the mice in the Der p group. However, there were no significant differences between the KWLL group and Der p group regarding collagen deposition levels (Figure 4(c)).

### 3.4. KWLL Affected T-Cell Subsets in BALF and Antibodies in Serum

**Figure 5:**
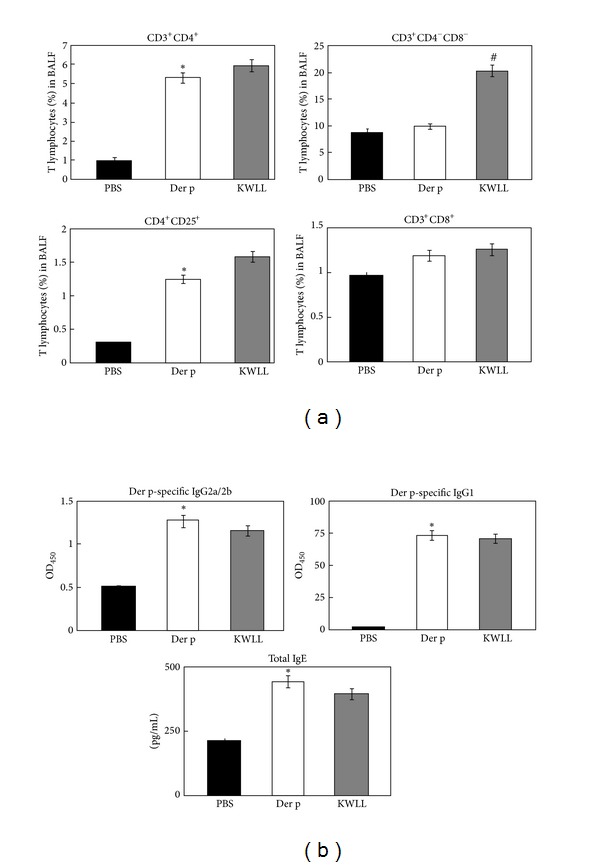
The T-cell subsets in BALF and the antibodies in the serum of challenged mice were affected by KWLL. (a) The immunofluorescence of monoclonal antibodies estimated the levels of CD3^+^CD4^+^, CD3^+^CD8^+^, CD4^+^CD25^+^, and CD3^+^CD4^−^CD8^−^ lymphocytes. (b) Total IgE and Der p-specific IgG1 and IgG2a/2b levels were measured by ELISA. Values represent the means ± SDs of 6 mice. *P < 0.05 (versus PBS group); ^#^ *P* < 0.05 (between nontreated and KWLL-treated groups).

We used monoclonal antibodies to determine by flow cytometry the influence of KWLL on T-cell subsets (Figure 5(a)). The results showed that the Der p group had more significantly increased percentages of CD3^+^CD4^+^ and CD4^+^CD25^+^ T cells than the PBS group. In contrast, the KWLL group mice showed no significant differences in the percentages of CD3^+^CD4^+^ and CD4^+^CD25^+^ T cells compared with the Der p group mice. However, the level of CD3^+^CD8^+^ T cells in BALF was not significantly different between the three groups of mice, but we found that the percentage of CD3^+^CD4^−^CD8^−^ (double negative) T cells in the KWLL group was higher than in the Der p group.

To evaluate the effect of KWLL on the humoral immune response status of repetitively Der p-challenged mice, sera were sampled and assayed for serum Der p-specific IgG1, IgG2a/2b and total IgE antibodies (Figure 5(b)). A significant enhancement in the levels of serum Der p-specific IgG1, IgG2a/2b and total IgE were observed in the Der p group mice compared with the levels in the PBS mice. However, the KWLL group showed no significant differences in the concentrations of Der p-specific IgG1, IgG2a/2b or total IgE compared with the Der p group.

### 3.5. KWLL Inhibited the Protein Production of Der p-Induced Proinflammatory and Cytokines in BALF

**Figure 6:**
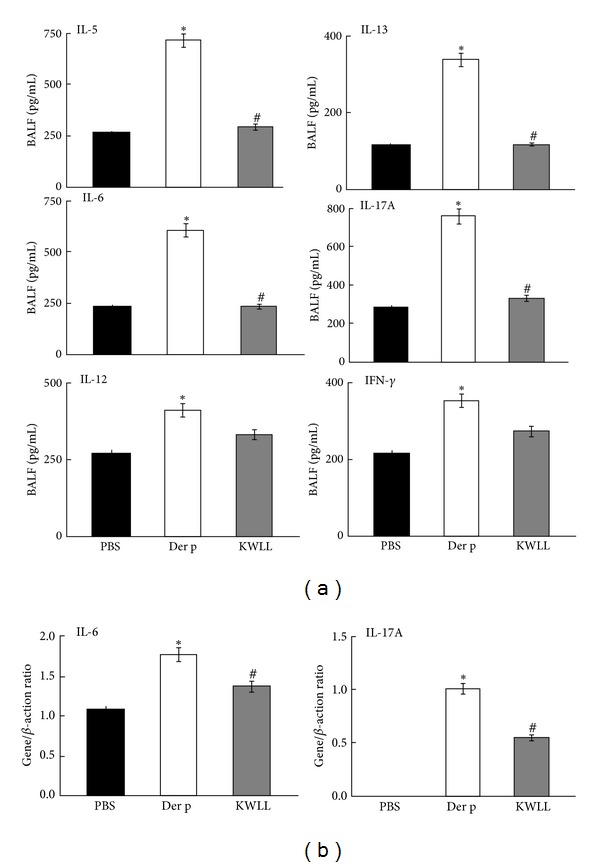
(a) Treatment of KWLL significantly reduced the protein products of IL-5, IL-6, IL-13, and IL-17A in the BALF of mice but did not affect the levels of IFN-*γ* and IL-12. Values represent the means ± SDs of 6 mice. (b) The gene expressions of IL-6 and IL-17A in lung tissue were measured. *P < 0.05 (versus PBS group); ^#^ *P* < 0.05 (between nontreated and KWLL-treated groups).

To analyze the presumed effect of KWLL on T-cells, we evaluated the influences of KWLL on T-cell cytokine secretion in BALF. The results showed that KWLL not only significantly attenuated the expression of Der p-induced T_H_2 cytokines, such as IL-5, IL-6, and IL-13 but also the T_H_17 cytokine like IL-17A than in the Der p group mice (Figure 6(a)). In addition, KWLL group mice slightly decreased the T_H_1 cytokines level of IFN-*γ* and IL-12 which were induced to overproduce by Der p than in the Der p group mice, although the differences in production were not significant.

### 3.6. KWLL Regulated the Gene Expression of Der p-Induced Proinflammatory Cytokines and Chemokines in Lung Tissues

The KWLL group showed significantly lower levels of IL-6 (down 0.62-fold) and IL-17A (down 0.51-fold) synthesis than the Der p group (Figure 6(b)). We found that the mice in the Der p group had markedly higher expression levels of MCP-1, eotaxin, and IL-1*β* than in the PBS group. However, the expression levels of IL-1*β*, eotaxin, and MCP-1 mRNA in the KWLL group slightly decreased than in the Der p group mice, but the differences in production were not significant (data not show). 

## 4. Discussion

In the present study, we found that KWLL treatment suppressed airway hyperresponsiveness and reduced airway inflammation cells in our mouse model of allergic asthma. There were significant reduction in total cell numbers and percentage of macrophages, neutrophils, and eosinophils in BALF of KWLL-treated group than those of Der p group. Moreover, treatment with KWLL also decreased the number of hyperplastic goblet cells and inflammatory cells, as shown by the lung histology analysis, except the degrees of collagen deposition. Therefore, the effect of KWLL on the reduced airway hyperresponsiveness in Der p allergen-sensitized and challenged mice might be associated with its action on the decreased inflammatory cells infiltration, such as eosinophils and neutrophils in the airways and goblet cells hyperplasia in the lung.

Recent investigations have reported the important role of T cells in the orchestration of the asthmatic inflammatory response making them as a potential clinical application for therapeutic targets [18–20]. In this study, we found that the number of lymphocytes in BALF was significantly increased in the oral KWLL-treated group compared with the Der p group (Figure 3). Treatment with KWLL did not have any effect on the isotype switch between T_H_1 and T_H_2 antibodies (Figure 5(b)). We speculated that oral KWLL could induce an increase in double-negative (CD3^+^CD4^−^CD8^−^) T cells and to a lesser degree CD25^+^CD4^+^ cells, but not the CD3^+^CD4^+^ and CD3^+^CD8^+^ T-cell subpopulations (Figure 5(a)). Hence, the immunomodulation effect of KWLL on allergen-sensitized mice might through the regulation and activation of T-cell subsets in the treated mice.

Eosinophils are the major effector cells in the initiation of the late phase of inflammation, and IL-5 has a specific role in eosinophil differentiation, growth, survival, and recruitment at inflammation sites [11, 21, 22]. Moreover, an increase in the airway hyperresponsiveness of asthma and airway remodeling may be due to increased production of IL-4, IL-5, IL-6, and IL-13 in the inflammated airways [23]. In present study, we showed that KWLL treatment decreased the levels of IL-5, IL-6, and IL-13 in BALF (Figure 6(a)). Therefore, through modulation of these T_H_2 cytokines, such as IL-5, IL-6, and IL-13, KWLL treatment was able to reduce eosinophils infiltration and airway hyperresponsiveness. However, KWLL treatment did not have any effect on the gene expression of eotaxin and MCP-1 (data not show), which were also related to eosinophils infiltration.

Elevated concentrations of IL-17A were found in allergic asthma patients, and this finding was correlated with the severity of asthma [24–27]. Recently, IL-17A has been shown to play a critical role in airway inflammatory, neutrophil recruitment, airway hyperresponsiveness, and airway remodeling [28], and it also enhances T_H_2 cell-mediated eosinophilic inflammation in the airways of patients with asthma [29]. IL-6 also plays a critical role in changing the balance between Treg and T_H_17 cells; thus, the regulation of IL-6 and IL-17 cytokines production and activities is potentially an effective application for the treatment of various autoimmune and inflammatory diseases [30]. In our study, KWLL treatment resulted in decreased secretion of IL-6 and IL-17A cytokines in BALF and downregulated mRNA expression of these cytokines in the lungs. These results indicate that KWLL inhibits neutrophil recruitment by modulating the T_H_17 balance but does not regulate the gene expression of IL-1*β* (data not show).

Moreover, our unpublished data also showed that there were dose-dependent effects in mice treated with increasing doses (one-, two-, and fourfold) of KWLL not only on the increased protein secretion and gene expression of IL-17A (data not shown) but also had serially enhanced airway hyperresponsiveness (Figure 2). Therefore, we speculate that KWLL reduces airway hyperresponsiveness by downregulating IL17, which is dose dependent. 

In conclusion, administering KWLL in chronically asthmatic mice increased T cells and reduced eosinophils and neutrophils recruitment into the lung. This immunoregulatory action might have resulted in a reduction of inflammation and airway hyperresponsiveness associated with the downregulation of IL-5, IL-6, and IL17. Therefore, this research provides the first evidence that KWLL has immunoregulatory effects on the remissive states of asthma. However, KWLL has not been characterized for its pharmacologically active components. Further research will be required to explore the molecular mechanisms of action of this TCM on chronic asthmatic patients.

## Abbreviations

BALF: Bronchoalveolar lavage fluid (s)
Der p: *Dermatophagoides*
i.t.: Intratracheal
TCM: Traditional Chinese medicine.
